# AI-driven hybrid rehabilitation: synergizing robotics and electrical stimulation for upper-limb recovery after stroke

**DOI:** 10.3389/fbioe.2025.1619247

**Published:** 2025-06-25

**Authors:** Ismail Ben Abdallah, Yassine Bouteraa, Ahmed Alotaibi

**Affiliations:** ^1^ Advanced Technologies in Medicine and Signals (ATMS), Ecole Nationale d’Ingénieurs de Sfax (ENIS), University of Sfax, Sfax, Tunisia; ^2^ Department of Computer Engineering, College of Computer Engineering and Sciences, Prince Sattam Bin Abdulaziz University, Al-Kharj, Saudi Arabia; ^3^ Department of Mechanical Engineering, College of Engineering, Taif University, Taif, Saudi Arabia; ^4^ King Salman Center for Disability Research, Riyadh, Saudi Arabia

**Keywords:** upper-limb rehabilitation, machine learning, electrical stimulation, muscle fatigue estimation, support vector machine (SVM), neuromuscular recovery

## Abstract

This study presents an AI-enhanced hybrid rehabilitation system that integrates a dual-arm robotic platform with electromyography (EMG)-guided neuromuscular electrical stimulation (NMES) to support upper-limb motor recovery in stroke survivors. The system features a symmetrical robotic arm with real-time anatomical adaptation for bilateral therapy and incorporates a Support Vector Machine (SVM)-based model for continuous muscle fatigue detection using time-frequency features extracted from EMG signals. A ROS2-based architecture enables real-time signal processing, adaptive control, and remote supervision by clinicians. The system dynamically adjusts stimulation parameters based on fatigue classification results, allowing personalized and responsive therapy. Preliminary clinical validation with three post-stroke patients demonstrated a 44% increase in range of motion, 45% enhancement in active torque, and 36% reduction in passive torque. The SVM model achieved a 95% accuracy in fatigue detection, and initial patient results suggest the feasibility and potential benefits of this intelligent, closed-loop rehabilitation approach.

## 1 Introduction

Rehabilitation is critical for restoring motor function in individuals affected by neurological disorders such as stroke, which often results in upper-limb impairments and reduced independence in daily living. In recent years, robotic rehabilitation systems have gained significant traction for their ability to deliver repetitive, high-intensity, and task-specific therapy—factors known to promote neuroplasticity and functional recovery (Kabir et al., 2022; Gopura et al., 2023). These systems offer distinct advantages over conventional therapy, including precise kinematic feedback, repeatable movement trajectories, and objective performance metrics (Carbone and Gonçalves, 2022; Han et al., 2023).

Various robotic modalities have emerged, including active-assisted devices, active-constrained robots, and adaptive exercise platforms, each tailored to patient-specific needs. Despite these advances, robot-only systems often lack the neuromuscular engagement necessary to restore voluntary motor control. To overcome this, hybrid rehabilitation strategies that combine robotic assistance with Functional Electrical Stimulation (FES) have been explored, leveraging the benefits of both modalities (Niu et al., 2022; Montoya et al., 2022).

Real-time control is a critical requirement in robotics, particularly in medical and rehabilitation applications where precision, safety, and responsiveness are paramount. The Robot Operating System 2 (ROS2) has emerged as a robust middleware framework designed to address real-time constraints and enhance modularity in robotic systems (Open Robotics). Despite its increasing adoption across various robotics domains, the application of ROS2 in rehabilitation robotics remains relatively limited, positioning our work among the pioneering efforts in this field. Research has demonstrated ROS2’s potential in rehabilitation applications, such as trajectory tracking for knee rehabilitation robots (Arcos et al., 2022) and brain–computer interface (BCI)-controlled robotic arms for assistive technology (Rivas et al., 2024). However, the broader implementation of ROS2 in rehabilitation exoskeletons and motor recovery systems is still emerging.

Recent advancements, including bioinspired hierarchical electronic architectures for robotic locomotion assistance (Delgado-Oleas et al., 2023) and the integration of digital twins with exoskeletons for telerehabilitation (Falkowski and Gwardecki, 2024), highlight the growing interest in intelligent, adaptable rehabilitation systems. Furthermore, IoT-enabled humanoid robotics for motor rehabilitation (Moghbelan et al., 2024) and multi-layered assessment approaches for hand spasticity using exoskeletons (Yu et al., 2025) reinforce the necessity of real-time, adaptive control frameworks. By leveraging ROS2’s enhanced communication reliability, reduced latency, and dynamic adaptability, our research contributes to expanding its applicability in medical robotics, demonstrating its potential to revolutionize patient-centered robotic interventions.

FES delivers electrical impulses to targeted muscles, inducing contractions that mimic voluntary movement. This approach has demonstrated efficacy in promoting motor relearning and upper-limb strength (Niu et al., 2019; Sousa et al., 2022; Tefertiller et al., 2022). However, traditional FES protocols are typically open-loop and static, often leading to overstimulation, patient discomfort, and premature muscle fatigue—particularly in patients with intact sensory pathways (Peckham and Knutson, 2005; Doucet et al., 2012). Moreover, most systems lack the ability to adapt stimulation parameters in real time based on muscle condition (Sharififar et al., 2018).

Machine learning (ML) has increasingly been applied to enhance adaptive neuromuscular electrical stimulation (NMES) for rehabilitation, offering personalized and precise control over functional electrical stimulation (FES). Recent advances in deep learning and ML-based FES controllers have demonstrated significant improvements in system adaptability, robustness, and patient-specific tuning. Researchers in Arcolezi et al. (2021) introduced an ML-driven approach to optimize the tuning of a robust integral of the sign of the error (RISE) controller for lower-limb rehabilitation, leveraging system identification techniques to improve NMES effectiveness. Similarly, research by Sierotowicz and Castellini (2023) explored robot-inspired human impedance control via functional electrical stimulation, applying ML-based techniques to adapt stimulation patterns dynamically based on user responses. These works underscore the growing role of intelligent controllers in rehabilitation, allowing for real-time adjustments and improved neuromuscular recovery. By integrating deep learning models into NMES control, future advancements can further enhance the precision and adaptability of rehabilitation protocols, ensuring optimal stimulation levels tailored to individual patients’ needs.

**FIGURE 1:**
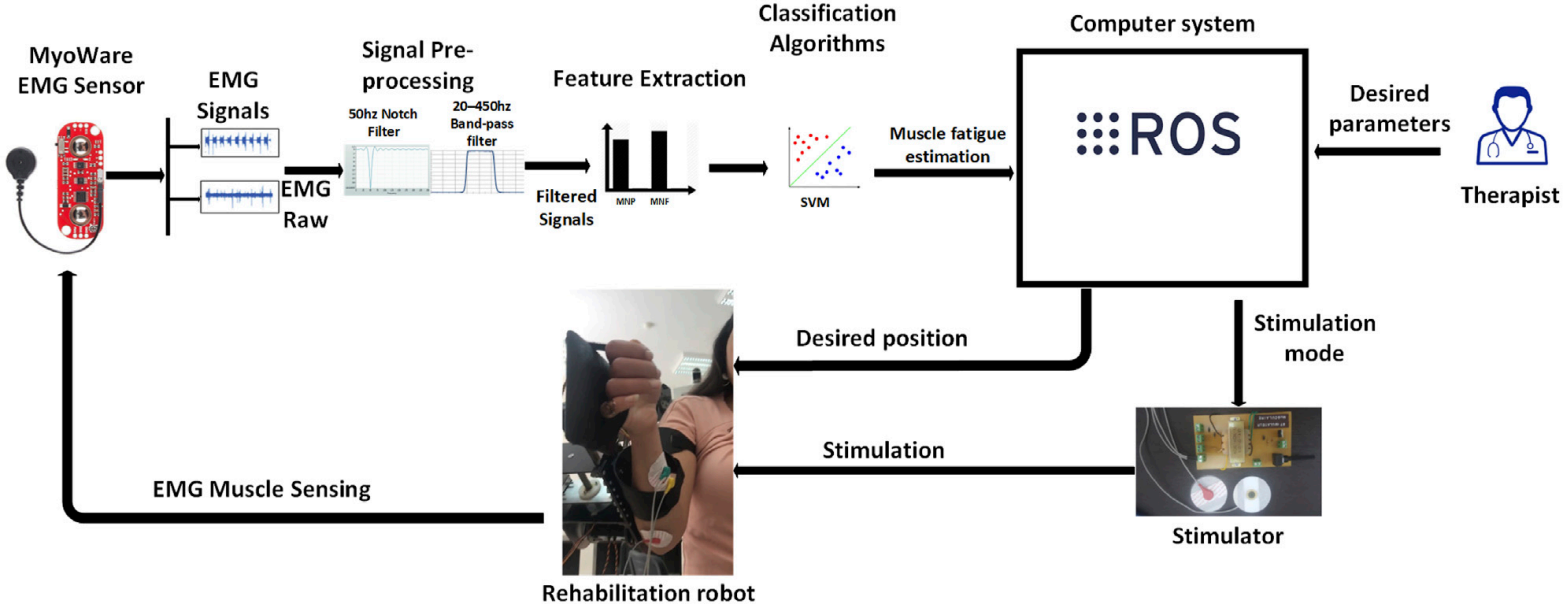
System overview illustrating the interaction between the robotic platform, EMG acquisition module, SVM-based fatigue estimation model, and stimulation control unit.

To address these limitations, we propose a closed-loop, AI-enhanced hybrid rehabilitation system that integrates robotic assistance with real-time, EMG-driven neuromuscular electrical stimulation, as illustrated in Figure 1. Central to our approach is a Support Vector Machine (SVM)-based model that continuously estimates muscle fatigue from EMG signals, enabling adaptive, patient-specific stimulation protocols. A symmetrical, dual-arm robotic platform was developed to support bilateral upper-limb therapy (Bouteraa et al., 2023; Bouteraa et al., 2020), featuring automatic limb-length adjustments to accommodate anatomical variations. The system is implemented using a ROS2-based control architecture, enabling real-time processing, modular extensibility, and remote therapist supervision.

This closed-loop configuration facilitates adaptive therapy sessions that dynamically respond to patient needs, ensuring both safety and effectiveness.

The main contributions of this work are as follows:

• Development of a dual-arm rehabilitation robot with real-time anatomical adaptation and bilateral therapy support.
• Integration of an EMG-driven adaptive stimulation protocol, using SVM-based fatigue classification for personalized intervention.
• Implementation of a ROS2-based control framework for real-time monitoring, configuration, and data acquisition.
• Preliminary Evaluationwith post-stroke patients, demonstrating measurable improvements in range of motion, neuromuscular activation, and torque output.

The remainder of this paper is structured as follows: Section 2 describes the system architecture and methodology. Section 3 presents experimental results and clinical analysis. Section 4 concludes with key findings and discusses future directions.

## 2 Materials and methods

### 2.1 Participants

Three male post-stroke patients (aged 48–66 years) with upper-limb motor impairments participated in this study. All patients were enrolled at the inpatient rehabilitation department of King Abdulaziz University Hospital in Jeddah, Saudi Arabia. The inclusion criteria included: (1) a history of ischemic or hemorrhagic stroke resulting in hemiparesis, (2) the ability to provide informed consent, and (3) absence of severe cognitive or sensory deficits.

The detailed clinical characteristics of the participants are summarized below:

• Patient 1: 52 years old, 14 months post-stroke, right-sided hemiparesis due to left middle cerebral artery (MCA) infarct, Fugl-Meyer Assessment Upper Extremity (FMA-UE) score = 28.
• Patient 2: 66 years old, 10 months post-stroke, left-sided hemiparesis due to right basal ganglia hemorrhage, FMA-UE = 35.
• Patient 3: 48 years old, 6 months post-stroke, right-sided hemiparesis due to left parietal infarct, FMA-UE = 22.

All participants presented with moderate upper-limb motor impairment and were undergoing post-acute rehabilitation. Ethical approval was obtained from the Institutional Review Board of King Abdulaziz University (IRB Ref: 34/22), and written informed consent was collected from all participants prior to enrollment.

All participants were in the chronic phase of stroke recovery (≥6 months post-onset), having completed their acute and early subacute rehabilitation phases prior to study enrollment. Clinical records and rehabilitation logs indicated minimal to no functional gains during the 4–8 weeks preceding the intervention, suggesting that natural recovery had largely plateaued. As such, the improvements observed during the study are unlikely to reflect spontaneous recovery alone and more likely stem from the hybrid robotic-stimulation intervention.

### 2.2 Rehabilitation robot design

The rehabilitation platform consists of a symmetrical dual-arm robotic system designed to deliver bilateral upper-limb therapy. Each arm includes a series of passive, low-friction joints connected via adjustable linkages to accommodate individual anatomical variations. The system is equipped with an automatic limb-length adaptation mechanism that ensures symmetrical alignment between the affected and unaffected arms.

The unaffected limb serves as a reference, guiding the motion of the impaired limb through synchronized bilateral movement. This configuration supports task-specific training, encourages motor relearning, and reduces the cognitive demand on the patient. The robot’s mechanical structure minimizes resistance and enables safe physical human-robot interaction.

**FIGURE 2:**
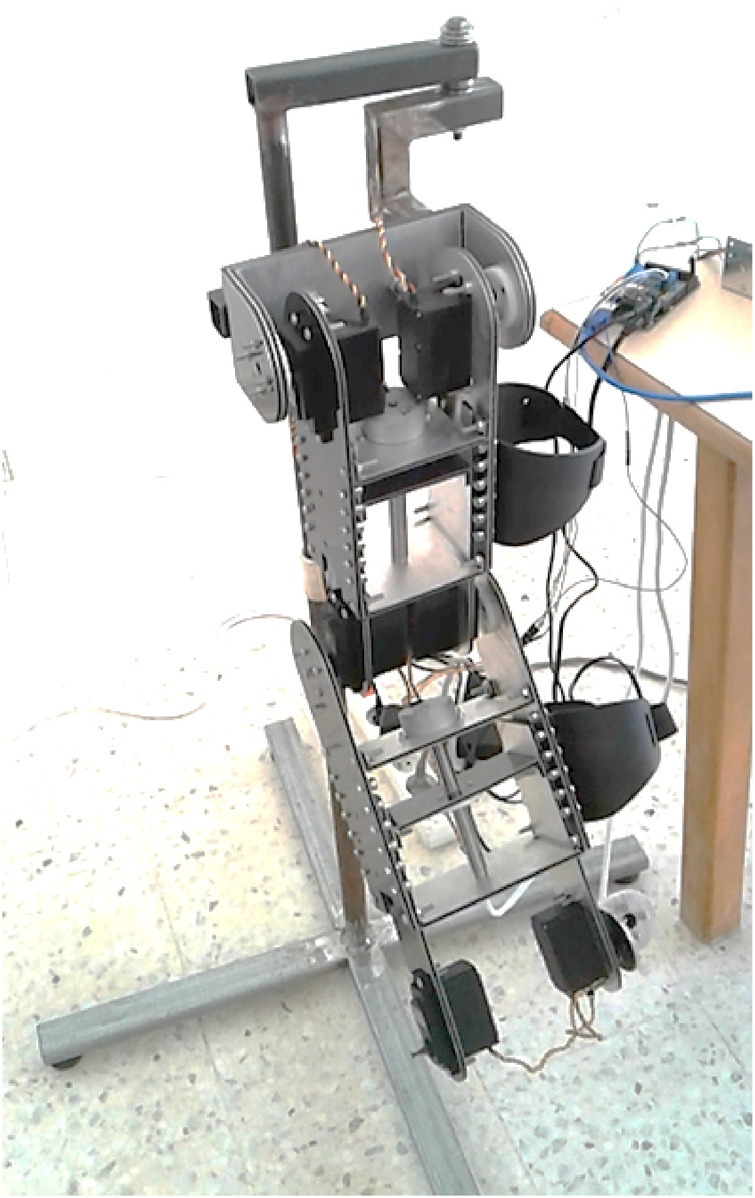
Mechanical design of the symmetrical dual-arm rehabilitation robot for bilateral training.

Figure 2 illustrates the complete setup of the dual-arm robot.

### 2.3 NMES integration

To enhance neuromuscular activation and support voluntary motor relearning, the robotic platform was integrated with a neuromuscular electrical stimulation (NMES) unit (Bouteraa et al., 2020). The NMES device delivers electrical pulses to specific muscles of the affected arm via surface electrodes. This configuration enables targeted stimulation of key muscle groups involved in the desired movements. The stimulation parameters—including pulse width, amplitude, and frequency—were dynamically adjusted in real time based on EMG-derived fatigue levels. When signs of fatigue were detected by the classification system, the stimulation intensity was modulated to reduce muscle overload and discomfort, ensuring continued engagement during therapy.

EMG signals were acquired from the affected limb using bipolar surface electrodes placed over the muscle belly and referenced to a neutral location. These signals were used not only for fatigue estimation, but also to guide the adaptive stimulation mechanism and monitor patient effort throughout the rehabilitation sessions.

This closed-loop integration ensures safe and personalized therapy by adapting stimulation in response to the patient’s real-time physiological state.

### 2.4 Control architecture

The control architecture of the hybrid rehabilitation system is built on the Robot Operating System 2 (ROS2) middleware (Open Robotics), enabling modular design, low-latency communication, and real-time signal processing. The software framework consists of three primary layers: data acquisition, signal processing and decision-making, and actuation.

In the first layer, raw EMG signals are continuously acquired through a USB interface and filtered to remove motion artifacts and ambient noise. These signals are then passed to the second layer, where time-domain and frequency-domain features are extracted. A pre-trained Support Vector Machine (SVM) classifier uses these features to estimate the level of muscle fatigue in real time.

Based on the output of the fatigue classifier, the third layer adjusts the parameters of the NMES device and commands the robotic actuators accordingly. ROS2 nodes handle synchronization between the data streams, publish EMG features, subscribe to classification outputs, and trigger stimulation adjustments. The system allows remote monitoring and configuration by clinicians via a user interface layer, promoting clinical usability and flexibility.

**FIGURE 3:**
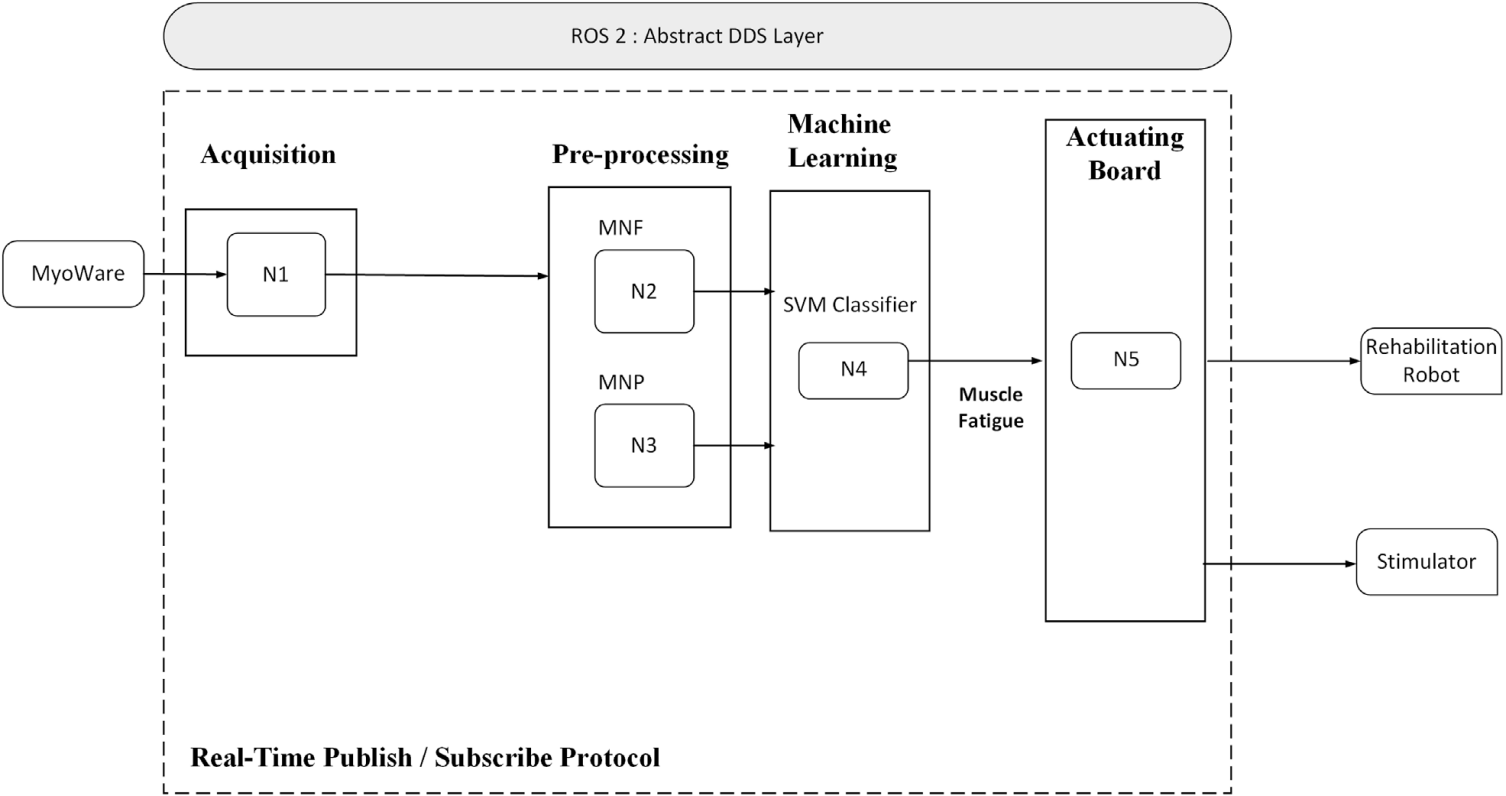
Overview of the ROS2-based control architecture integrating EMG processing, fatigue detection, and stimulation control.

Figure 3 provides an overview of the ROS2-based control loop, showing the integration of biosignal acquisition, processing, and adaptive actuation.

### 2.5 EMG signal processing

The EMG signals were acquired from specific muscles of the affected arm using bipolar surface electrodes. Signals were sampled at 1 kHz and transmitted via a USB interface to the ROS2 processing unit. Pre-processing steps included a fourth-order Butterworth band-pass filter (20–450 Hz) to remove motion artifacts and power line interference, followed by full-wave rectification and smoothing using a moving average window of 200 ms. These operations reduced noise and prepared the signals for feature extraction.

A set of time-domain and frequency-domain features were extracted from overlapping signal windows with a step size of 100 ms. The selected features included:

• Root Mean Square (RMS) – estimates signal energy;
• Mean Absolute Value (MAV) – reflects muscle contraction level;
• Zero Crossing (ZC) – relates to frequency and signal variability;
• Mean Frequency (MF) – indicates muscle fatigue;
• Mean Power (MP) – provides insight into muscle activation intensity.

These features were chosen based on their sensitivity to muscle fatigue and classification performance (Sun et al., 2022; Phinyomark et al., 2012). The extracted feature vectors served as input to the SVM model described in Section 2.6.

Although our signal preprocessing pipeline involved rectification and smoothing—steps which may attenuate high-frequency components—we verified that the features retained strong discriminative power. To illustrate this, we applied Principal Component Analysis (PCA) to the feature space and projected the data onto the first two principal components. As shown in Figure 9, the Fatigue and Non-Fatigue classes formed distinguishable clusters, supporting the validity of our preprocessing approach. This confirms that, despite the non-standard filtering method, the extracted features remain informative for classification tasks.

### 2.6 SVM classification and fatigue estimation

To detect muscle fatigue in real time, a supervised Support Vector Machine (SVM) model was developed using features extracted from EMG signals as described in Section 2.5. The classification problem was formulated as a binary task: distinguishing between Fatigue and Non-Fatigue states based on changes in muscle signal characteristics.

Ground truth labels for fatigue states were generated using a dual-criterion method based on both mechanical output and EMG signal characteristics. First, a segment was marked as “Fatigue” if there was a decline of more than 20% in active torque relative to the session baseline, indicating reduced muscular performance. Second, the corresponding EMG segment had to show a decrease of at least 10% in mean frequency (MF), reflecting muscle fatigue-related spectral shifts. Only when both conditions were simultaneously met was the data labeled as “Fatigue.” Segments that did not satisfy both thresholds were labeled as “Non-Fatigue.” Although subjective patient feedback and clinician input were not incorporated in this pilot phase, we recognize their potential for enhancing labeling accuracy in future studies.

The selected features (RMS, MAV, ZC, MF, and MP) were normalized and used to train the SVM model using a radial basis function (RBF) kernel.

To train and evaluate the SVM classifier, we pooled labeled EMG data from all three participants and applied stratified 5-fold cross-validation. This ensured balanced representation of fatigue and non-fatigue samples in each fold. The model achieved an average classification accuracy of 95%. However, we recognize that pooling data across subjects may result in overly optimistic performance due to intra-subject data leakage. This limitation is particularly relevant when the goal is to generalize to new, unseen patients. In future work, we plan to implement leave-one-subject-out (LOSO) cross-validation to better assess subject-independent generalization and refine model robustness accordingly.

### 2.7 Adaptive FES control algorithm

The electrical stimulation parameters were dynamically adapted based on the fatigue classification results from the SVM model. Specifically, the SVM classified muscle fatigue into three categories: Low Fatigue, Moderate Fatigue, and High Fatigue. Each fatigue level was associated with a predefined stimulation amplitude range:

• Low Fatigue: 25–30 mA
• Moderate Fatigue: 18–24 mA
• High Fatigue: 12–17 mA

When a new fatigue level was detected, the control system smoothly transitioned to the corresponding stimulation range using linear ramping over a 3–5 s window to avoid abrupt intensity changes. To prevent oscillatory behavior due to classification fluctuations, a hysteresis threshold of 10% in fatigue feature metrics was implemented. Additionally, transitions were rate-limited to no more than one profile change per 15 s. This ensured system stability, reduced patient discomfort, and maintained effective muscle engagement throughout the session.

**FIGURE 4:**
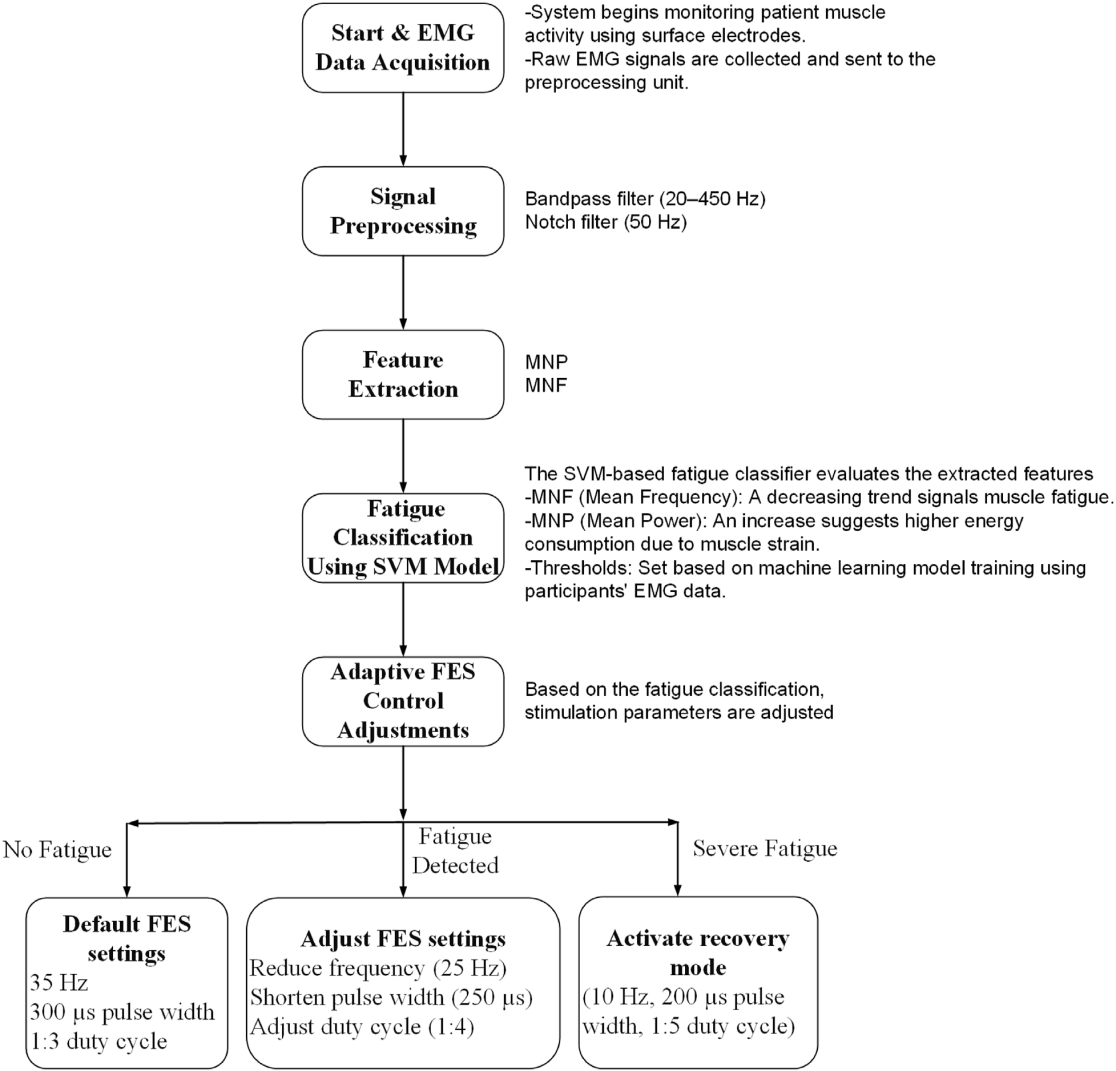
Operating flowchart showing real-time fatigue detection and adaptive stimulation control. The terms “Fatigue Detected” and “Severe Fatigue” correspond to the Moderate and High Fatigue states, respectively, as determined by SVM decision thresholds.

As shown in Figure 4, the system continuously monitored EMG signals during exercise. When the SVM classifier detected a change in fatigue level, the controller adjusted the stimulation amplitude accordingly and updated the ROS2 topic communicating with the NMES unit. Transitions between the three stimulation profiles—Low Fatigue, Moderate Fatigue, and High Fatigue—were based on the decision function output of the SVM classifier. Specifically, we used crisp thresholds on the SVM’s signed distance from the separating hyperplane.

• Low Fatigue: decision value > +0.6
• Moderate Fatigue: −0.6 ≤ decision value ≤ +0.6
• High Fatigue: decision value < −0.6

These thresholds were empirically selected to ensure consistent performance during pilot sessions. To prevent frequent state switching due to borderline values, a hysteresis mechanism was implemented: transitions between adjacent fatigue states required sustained decision values beyond the boundary threshold for at least two consecutive windows (200 ms each). This approach provided stable, interpretable control and enabled smooth modulation of stimulation intensity.

This adaptive approach allowed the stimulation to remain personalized and responsive to the patient’s physiological state throughout the rehabilitation sessions, reducing the risk of discomfort and maximizing functional engagement.

### 2.8 Experimental protocol

Each participant underwent a 6-week hybrid rehabilitation program composed of 30 therapy sessions (five sessions per week). Each session lasted approximately 45 min and included a warm-up phase, repeated training cycles, and short rest intervals. The therapy focused on task-specific, symmetrical bilateral movements facilitated by the robotic system and guided by the stimulation controller.

At the beginning of each session, baseline EMG activity was recorded during a relaxation period to calibrate the classifier. During active training, the system monitored muscle activity and fatigue in real time, adjusting the NMES intensity accordingly. Participants were seated in an upright position, and arm alignment with the robot was verified before each session.

A trained therapist supervised the sessions and intervened when necessary to assist or adjust stimulation parameters. All patient responses, signal trends, and system logs were recorded for offline analysis.

**FIGURE 5:**
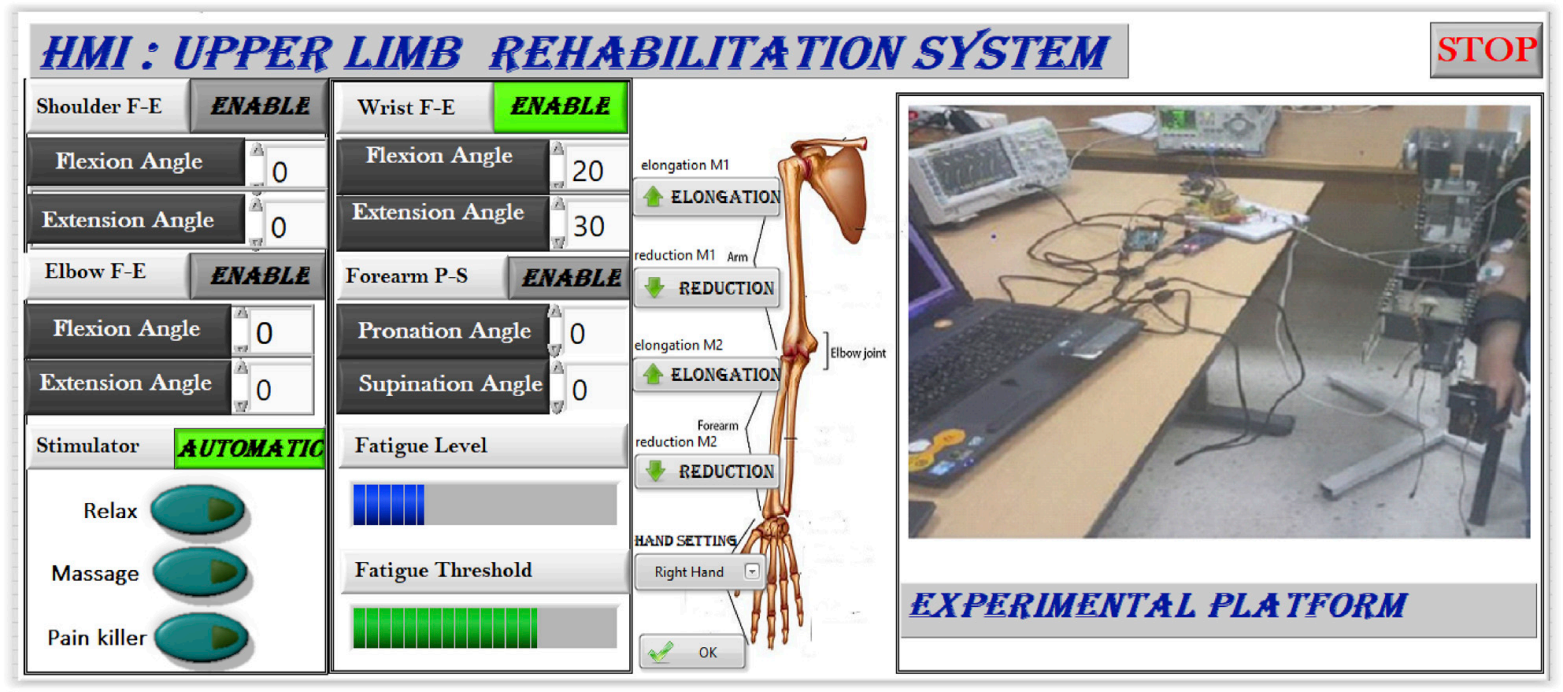
Experimental protocol: hybrid system setup with patient during a supervised therapy session.

Figure 5 shows the experimental setup used during the therapy sessions.

The Rehabilitation Progress Factor (RPF) is a simplified, intuitive metric designed to summarize each patient’s motor improvement in terms of joint flexibility. It is defined as the ratio between the final achieved range of motion (RoM) and a clinically desired or target RoM, normalized to a 0–10 scale (Equation 1):

$$RPF=\left(\frac{{RoM}_{post}}{{RoM}_{target}}\right)\times 10$$ (1)

The target RoM was set to 90°, based on clinical guidelines for functional upper-limb mobility. For example, a patient who achieved 82° of RoM at the end of the intervention would receive an RPF score of approximately 9.1. This score provides an intuitive summary of joint mobility progress, aiding clinicians in evaluating the effectiveness of therapy and guiding further interventions.

**FIGURE 6:**
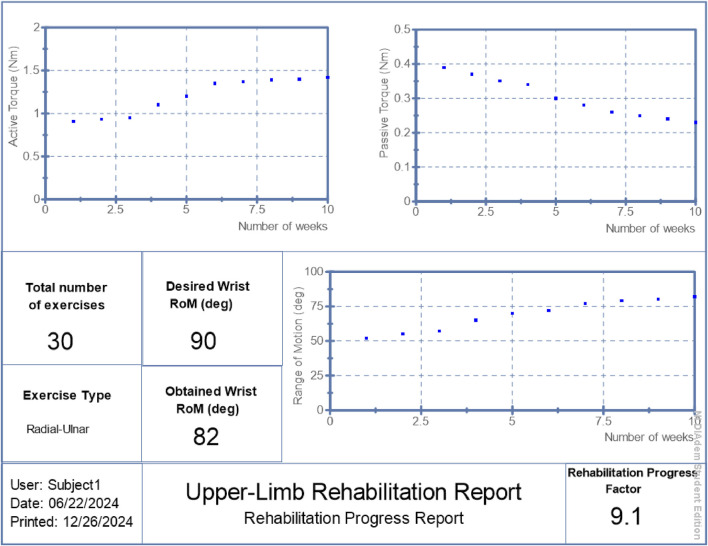
Generated report: patient 1.

**FIGURE 7:**
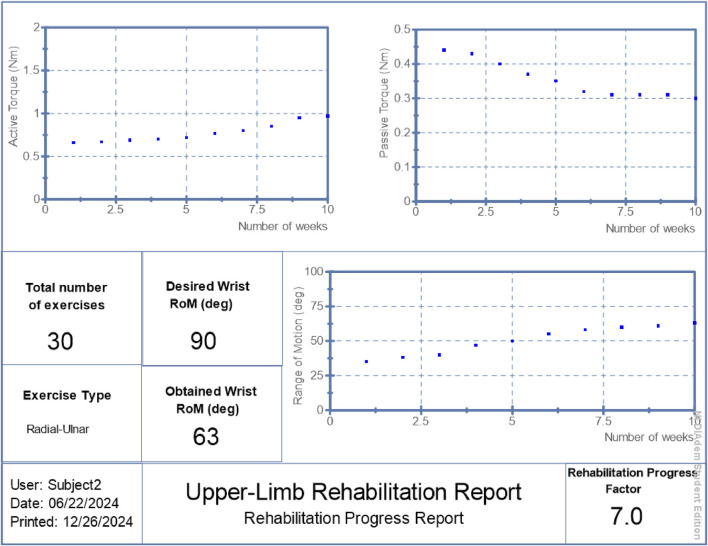
Generated report: patient 2.

**FIGURE 8:**
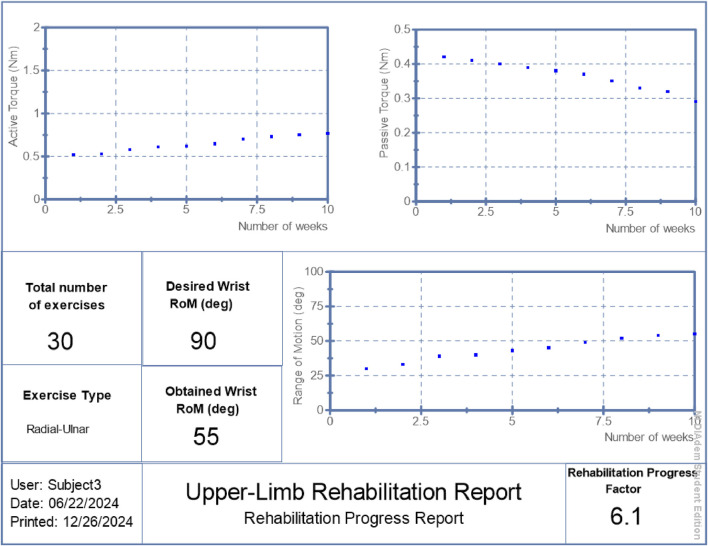
Generated report: patient 3.

RPF values were reported in Figures 6–8 alongside torque and motion metrics to offer a high-level view of recovery.

## 3 Results

The proposed hybrid rehabilitation system was evaluated on three post-stroke patients over 30 therapy sessions conducted across a 6-week period. Quantitative assessments were carried out before and after the intervention, focusing on three clinical metrics: range of motion (RoM), active torque, and passive torque at the targeted joint. As shown in Figures 6–8, the system enabled substantial improvements across all three patients. On average:

• Range of motion increased by 44%, reflecting enhanced joint flexibility.
• Active torque increased by 45%, indicating improved voluntary muscular activation.
• Passive torque decreased by 36%, showing reduced joint resistance and stiffness.

To further validate system performance at an individualized level, automated reports were generated by the system’s ROS2 interface, summarizing patient-specific progress over time. These are presented in Figures 6–8, respectively.

The summarized outcomes were:

• Patient 1: Final RoM of 82°, RPF = 9.1/10, active torque increased from 0.91 Nm to 1.42 Nm, passive torque reduced from 0.39 Nm to 0.23 Nm.
• Patient 2: Final RoM of 63°, RPF = 7.0/10, active torque reached 0.97 Nm, passive torque reduced to 0.30 Nm.
• Patient 3: Final RoM of 55°, RPF = 6.1/10, active torque increased to 0.77 Nm, passive torque lowered to 0.29 Nm.

These individualized reports suggest the system’s potential to support measurable functional improvements while adapting stimulation to real-time physiological states. Although the improvements in range of motion, active torque, and passive torque were consistent across all patients, the small sample size (n = 3) limited statistical power. Wilcoxon signed-rank tests were performed and yielded p-values of 0.25 across all three metrics. While these values did not reach conventional significance thresholds, the observed trends suggest meaningful clinical improvement and motivate larger-scale studies for future validation.

**FIGURE 9:**
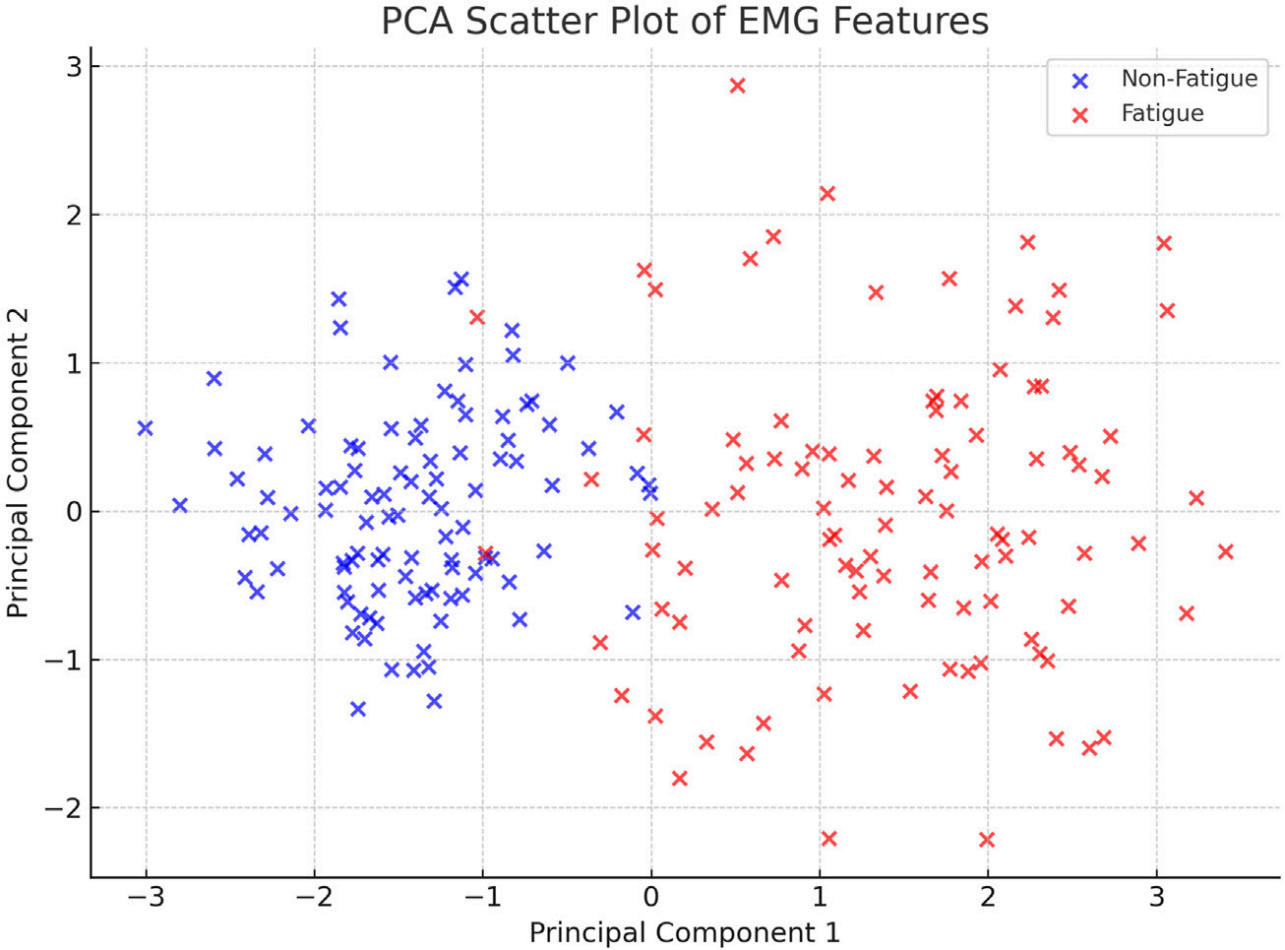
PCA-based scatter plot of extracted EMG features. The projection onto the first two principal components reveals a clear separation between the Fatigue and Non-Fatigue classes, supporting the effectiveness of the preprocessing and feature extraction pipeline.

Figure 9 presents the PCA scatter plot of the EMG features. As shown, there is a clear separation between the Fatigue and Non-Fatigue classes in the space of the first two principal components, validating the discriminative quality of the features despite the preprocessing steps.

## 4 Discussion

The results of this pilot study suggest that the integration of a symmetrical dual-arm robotic platform with adaptive, EMG-driven neuromuscular stimulation can effectively enhance upper-limb rehabilitation outcomes in post-stroke patients. Specifically, we observed measurable improvements in range of motion, increased active torque, and decreased passive resistance across all participants. These gains can be attributed to three key factors: (1) the use of bilateral symmetry training, which leverages mirrored motion to promote inter-limb coordination and neuroplasticity; (2) the implementation of a fatigue-aware stimulation strategy, which maintains engagement while preventing overstimulation; and (3) the adoption of a real-time, ROS2-based control system, which ensures responsive adaptation and continuous clinician oversight. Together, these components create a synergistic framework that enhances therapy personalization and efficacy. Improvements in range of motion, active torque, and passive torque highlight both neuromuscular reactivation and reduction of joint stiffness. The range of motion (ROM) in the joints of the three participants increased by approximately 44% from their initial states, demonstrating the effectiveness of the rehabilitation robot in enhancing joint mobility. These results are broadly consistent with recent trends reported in the literature (Rouse et al., 2020; Mashayekhi and Moghaddam, 2022), where comparable systems have achieved 30%–40% improvements in RoM over similar rehabilitation durations. While our observed ∼44% improvement is encouraging, caution is warranted in making direct comparisons due to differences in sample size, patient characteristics, and evaluation protocols.

Similarly, joint torque assessments revealed a 45% increase in active torque and a 36% reduction in passive torque, highlighting the system’s ability to improve neuromuscular function. These outcomes represent a significant advancement compared to our previous work using exoskeletons alone (Bouteraa et al., 2023) and traditional electrical stimulation techniques that did not consider muscle fatigue (Bouteraa et al., 2020). Related studies have combined exoskeletons with functional electrical stimulation (Inoue et al., 2022; Abdallah and Bouteraa, 2024), achieving moderate improvements in motor function. However, the integration of real-time muscle fatigue classification in our proposed protocol enables more adaptive and personalized interventions, leading to superior rehabilitation outcomes.

While recent research has investigated muscle fatigue estimation during rehabilitation training (Mashayekhi and Moghaddam, 2022; Zasadzka et al., 2022; Maffiuletti et al., 2018), integrating fatigue information directly into the primary control system remains a considerable challenge. In most existing systems, patient safety is managed manually by pausing rehabilitation sessions or modifying stimulation settings when signs of fatigue are observed.

This study addresses that limitation by implementing a robust Support Vector Machine (SVM) classifier capable of detecting muscle fatigue with a mean accuracy of 95%, outperforming previous approaches (Zasadzka et al., 2022) where fatigue detection accuracy was limited to 90% using linear models.

Unlike traditional open-loop stimulation systems that apply fixed parameters throughout therapy, our approach adapts stimulation in real time based on muscle fatigue levels estimated via EMG signals and SVM classification. This closed-loop mechanism allows the system to optimize therapeutic intensity without exceeding fatigue thresholds—improving comfort and engagement. Similar adaptive strategies have been explored in exoskeleton control (Montoya et al., 2022), but few have combined fatigue adaptation with bilateral robotic training and ROS2 integration.

The system’s ability to generate patient-specific reports—including real-time tracking of torque and mobility parameters—offers a significant advantage for clinical monitoring and decision-making. The inclusion of the RPF adds a high-level indicator of recovery progress, useful for both therapists and automated systems.

Bilateral mirrored motion, achieved through the symmetrical robot design, provides consistent proprioceptive feedback and leverages inter-limb coordination to stimulate neuroplasticity. Coupled with adaptive FES, this synergy supports the re-establishment of functional motor pathways (Niu et al., 2022).

Despite promising outcomes, the study is limited by a small sample size. Future work will include a larger clinical cohort, integration of cognitive intent (via EEG), and long-term outcome tracking. Nevertheless, the results demonstrate the viability and therapeutic potential of a real-time, AI-enhanced hybrid rehabilitation system grounded in physiological data. Another limitation of this pilot study is the absence of standardized clinical outcome assessments, such as the Action Research Arm Test (ARAT) or post-intervention Fugl-Meyer scores. Although objective biomechanical measures (e.g., RoM, torque) provided valuable insights into neuromuscular improvements, the inclusion of validated clinical scales would enhance the clinical relevance and comparability of results. Future studies will integrate such tools to provide a more comprehensive evaluation of functional recovery.

## 5 Conclusion

This study presented a novel AI-driven hybrid rehabilitation system that integrates symmetrical dual-arm robotic assistance with adaptive neuromuscular electrical stimulation, guided by real-time EMG analysis and machine learning. The proposed framework leverages a Support Vector Machine classifier to detect muscle fatigue and dynamically adjust stimulation intensity, ensuring patient specific, fatigue-aware therapy.

Preliminary testing on three post-stroke patients demonstrated measurable improvements in range of motion, active torque, and reduction in joint stiffness. In addition to group-level gains, individualized reports highlighted the system’s ability to adaptively track and optimize progress in real time. The ROS2-based control architecture enabled seamless integration of sensing, processing, actuation, and reporting—facilitating modularity, clinical usability, and remote supervision. These promising findings motivate further investigation with larger clinical trials to robustly evaluate efficacy and generalizability.

By addressing key limitations of conventional FES and robot-only systems, this work contributes a scalable, closed-loop solution for next-generation neurorehabilitation. Future efforts will focus on expanding clinical trials, incorporating EEG-based cognitive intent detection, and further enhancing the system’s autonomy and personalization.

## Data Availability

The original contributions presented in the study are included in the article/supplementary material, further inquiries can be directed to the corresponding author.
